# Supplementary material for: Extended sample size calculations for evaluation of prediction models using a threshold for classification
Source: BMC Med Res Methodol. 2025 Jul 1;25:170. doi: 10.1186/s12874-025-02592-4 (PMC12210805; doi:10.1186/s12874-025-02592-4)
Supplement: Supplementary file 1 — Supplementary Material 1. [file 12874_2025_2592_MOESM1_ESM.docx]

**Expansion of Eq.(5)**

Replacing $cov\left( P,R \right)$ in Eq.(3) with Eq.(4) gives us:

$$SE_{F1}=\sqrt{4\times\frac{R^{4}SE_{P}^{2}+2P^{2}R^{2}\left( \frac{\frac{P\left( 1-P \right)\left( 1-R \right)}{\emptyset}+\frac{P\left( 1-P \right)\times Specificity}{1-\emptyset}}{N} \right)+P^{4}SE_{R}^{2}}{\left( P+R \right)^{4}}.}$$

By squaring the above equation, we get:

$$SE_{F1}^{2}=4\times\frac{R^{4}SE_{P}^{2}+2P^{2}R^{2}\left( \frac{\frac{P\left( 1-P \right)\left( 1-R \right)}{\emptyset}+\frac{P\left( 1-P \right)\times Specificity}{1-\emptyset}}{N} \right)+P^{4}SE_{R}^{2}}{\left( P+R \right)^{4}},$$

which can be rearranged to give:

$$\frac{\frac{SE_{F1}^{2}\left( P+R \right)^{4}}{4}-R^{4}SE_{P}^{2}-P^{4}SE_{R}^{2}}{2P^{2}R^{2}}=\frac{\frac{P\left( 1-P \right)\left( 1-R \right)}{\emptyset}+\frac{P\left( 1-P \right)\times Specificity}{1-\emptyset}}{N}.$$

Therefore,

$$N=\frac{2P^{2}R^{2}\left( \frac{P\left( 1-P \right)\left( 1-R \right)}{\emptyset}+\frac{P\left( 1-P \right)\times Specificity}{1-\emptyset} \right)}{\left( \frac{SE_{F1}^{2}\left( P+R \right)^{4}}{4} \right)-R^{4}SE_{P}^{2}-P^{4}SE_{R}^{2}}$$
